# Incidental Discovery of Malignant Pheochromocytoma During Preoperative Coronary Artery Bypass Surgery (CABG) Workup

**DOI:** 10.7759/cureus.97075

**Published:** 2025-11-17

**Authors:** Christian Summa, Kelcie Lushefski, Jacqueline C Oxenberg

**Affiliations:** 1 General Surgery, Geisinger Wyoming Valley Medical Center, Wilkes-Barre, USA; 2 Endocrinology, Mayo Clinic, Rochester, USA; 3 Surgical Oncology, Lehigh Valley Cancer Institute, East Stroudsburg, USA

**Keywords:** adrenal mass, catecholamines, coronary artery disease, neuroendocrine tumor, pheochromocytoma, pre operative evaluation

## Abstract

Pheochromocytomas are catecholamine-secreting neuroendocrine tumors that occur in less than 0.2% of patients presenting with hypertension. Of these, approximately 10% are found to have malignant potential, thus further demonstrating their rarity. In patients with uncontrolled hypertension, a pheochromocytoma should be included in the differential diagnosis.

A 54-year-old male with a past medical history of coronary artery disease (CAD) and hypertension presented to the emergency department with chest pain. On workup, there was concern for acute coronary syndrome, and he was taken to the cardiac catheterization suite. He was found to have multi-vessel CAD, and the decision was made for a coronary artery bypass surgery (CABG). Prior to the procedure, he was found to have a left adrenal mass, which was ultimately found to be a malignant pheochromocytoma.

Pheochromocytomas are rare neuroendocrine tumors composed of chromaffin cells, which are responsible for the secretion of catecholamines. These tumors occur in less than 0.2% of patients with hypertension and can be benign or malignant, with malignancy occurring in 10% of cases. According to the World Health Organization (WHO), malignant pheochromocytomas can only be defined with the presence of regional invasion or metastasis in a non-chromaffin site.

In conclusion, despite the rarity of pheochromocytomas, it is important to keep these tumors in the differential diagnosis, especially when considering a patient with refractory hypertension. Any incidentaloma with appropriate clinical history, such as uncontrolled hypertension, should raise the suspicion of pheochromocytoma.

## Introduction

We present a patient who was found to have an incidentaloma during pre-operative workup for a coronary artery bypass surgery (CABG), which was later found to be a malignant pheochromocytoma. Pheochromocytomas are catecholamine-secreting neuroendocrine tumors that occur in less than 0.2% of patients presenting with hypertension (HTN). Although rare, given the potential for malignancy, they should not be ignored when discovered. Below we describe a patient with a 2 x 3 cm left incidentaloma and multiple failed antihypertensive medications who was diagnosed with a malignant pheochromocytoma.    

## Case presentation

**Figure 1:**
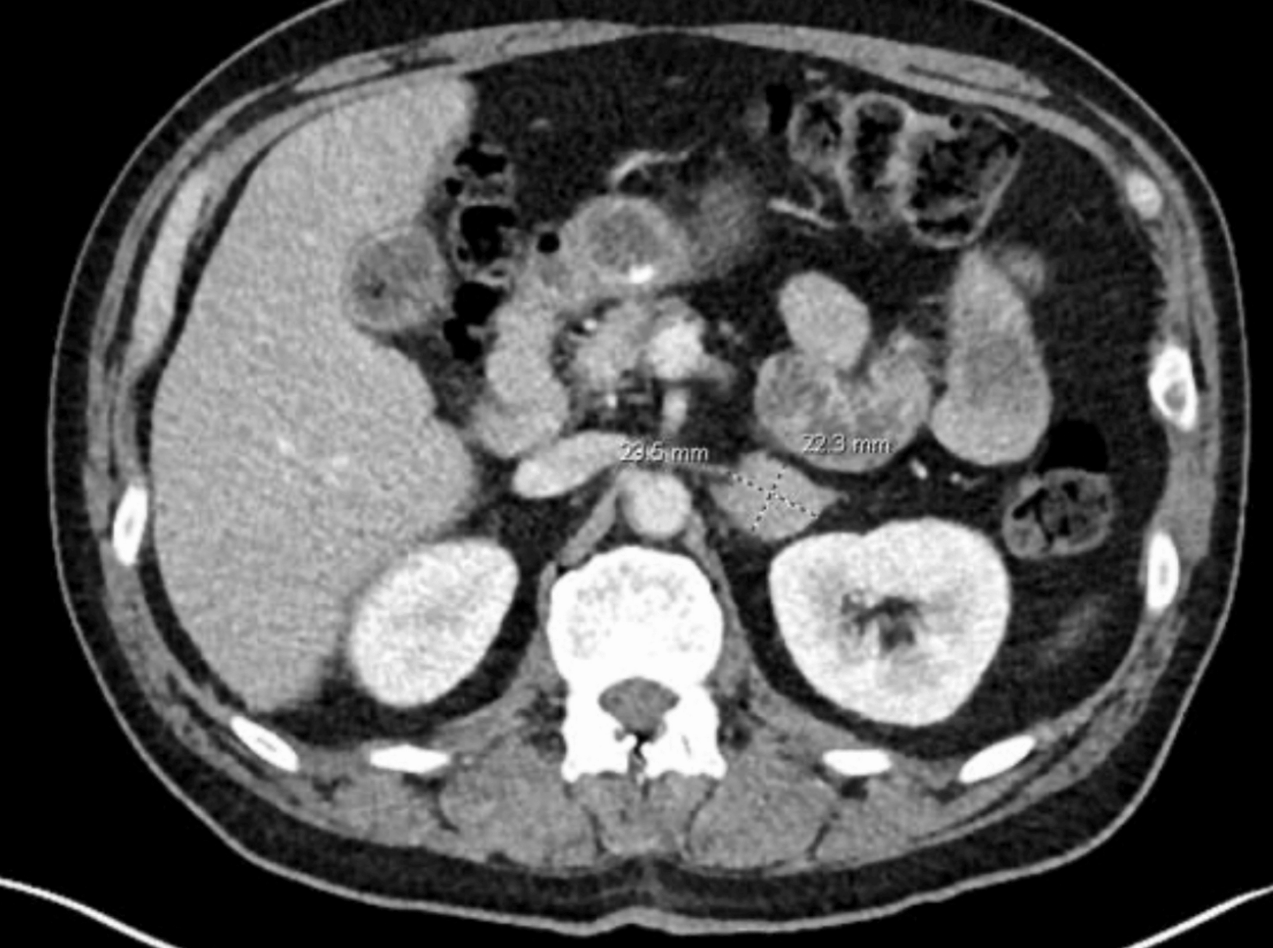
Axial section of CT abdomen with intravenous contrast showing a 2.2 x 2.4 cm left adrenal mass.

A 54-year-old male with a past medical history of coronary artery disease (CAD) and HTN presented to the emergency room with chest pain. On workup in the emergency room, there was concern for acute coronary syndrome (ACS), and he was taken to the cardiac catheterization suite immediately. He was found to have multivessel CAD, and it was decided that he undergo CABG in the near future. The workup in the emergency room included a computed tomography (CT) of the chest, abdomen, and pelvis.  He was found to have a 2 x 2.4 cm left adrenal mass as shown in Figure 1. The patient was referred to the surgical oncology office, per further chart review revealed that the adrenal mass had been known to the patient for about six years and was being followed by urology. At the appointment the patient also admitted to uncontrolled HTN, requiring four antihypertensives in the past. Biochemical testing was ordered and his normetanephrines were noted to be 1444 nmol/L (normal range 44-261 nmol/24 hours). At this time, there was a concern for a pheochromocytoma, and he was started on phenoxybenzamine, an alpha-adrenergic blocker, prior to his CABG. One month after his CABG, he underwent robotic-assisted left adrenalectomy with intraoperative ultrasound. His pathology returned as a 4.7 cm pheochromocytoma with features suggestive of aggressive behavior due to vascular invasion, capsular invasion, invasion of periadrenal adipose tissue, and 4+ mitotic figures per 10 high-powered fields with a Pheochromocytoma of Adrenal Gland Scaled Score (PASS) of 6. He then underwent adjuvant radiation, and one month after surgery, his metanephrines trended down from 1444 to 262 nmol/L. Due to concern over the continued elevation in metanephrines, a Netspot scan was performed. The Netspot scan was negative for residual or recurrent disease, and the patient continues to be disease-free.   

## Discussion

Pheochromocytomas are rare neuroendocrine tumors composed of chromaffin cells, which are responsible for the secretion of catecholamines [1]. These tumors, which are often discovered incidentally, occur in less than 0.2% of patients with hypertension and present equally in both men and women. Typically, the diagnosis is made between the third and fifth decade of life [1]. These tumors can be sporadic or familial with up to 30% of pheochromocytomas having a genetic disposition. These syndromes with a genetic disposition include von Hippel-Lindau (VHL), multiple endocrine neoplasia type 2 (MEN2), and neurofibromatosis 1 (NF1).    

Pheochromocytomas can be benign or malignant, with malignancy occurring in 10% of cases. Both benign and malignant tumors are histologically and biochemically the same. Local tissue invasion to the surrounding tissues is the most reliable indicator of metastatic disease. Symptoms of pheochromocytomas are typically caused by the hypersecretion of catecholamines (norepinephrine, epinephrine, or dopamine), thereby increasing central sympathetic activity. The classic triad of symptoms includes sweating, tachycardia, and episodic headaches. Interestingly, Young et al. found that most patients with pheochromocytomas do not present with these three classic symptoms. Instead, sustained or paroxysmal hypertension was found to be the most common presenting symptom, although not present in 5-15% of patients. Thus, strong emphasis is placed on obtaining a proper history and physical exam for all potential signs and symptoms, as in our patient, who was found to have known about this incidentiloma. Initial biochemical investigation includes plasma and/or urine catecholamines and metanephrine levels [1]. Selection of testing is typically dependent on level of suspicion. Young et al. suggest 24-hour urinary fractionated catecholamines and metanephrines for patients with a lower index of suspicion, and plasma fractionated metanephrines for definitive diagnosis for patients with a higher index of suspicion [2]. Another way to diagnose pheochromocytomas is on imaging. Approximately 60% of patients with pheochromocytomas are discovered incidentally during CT (>10 Hounsfield units and marked enhancement with IV contrast) or magnetic resonance imaging (MRI) (high signal intensity on T2-weighted MRI or cystic/hemorrhagic appearance) for non-related symptoms [2]. As presented in this case, it is important to discuss with patients when incidentalomas are discovered on imaging.    

Once a pheochromocytoma has been identified and diagnosed, the tumor should be resected [3]. Preoperative optimization, including close monitoring of cardiovascular and hemodynamic changes, is imperative prior to surgical resection. Administration of preoperative alpha blockers at least seven days prior to surgery allows for control of blood pressure and intravascular expansion [1]. The importance of pre-operative optimization is clinically evident as patients with undiagnosed pheochromocytomas have significantly higher surgical mortality rates due to the potential of lethal hypertensive crisis, arrhythmias, and multiorgan failure [4]. Surgery for benign tumors has been successful with a five-year survival rate of 95% [1]. In recent studies, O’Dwyre et al. demonstrate how the laparoscopic approach is preferential over an open operation secondary to easier access as well as benefits of the magnification view [5].   

Standardizing diagnostic methods for metastatic pheochromocytomas is a challenge that has yet to be answered. The five-year survival rate for a benign pheochromocytoma is 95%; however, the five-year survival rate for a malignant pheochromocytoma is nearly 50% [4]. Clinical and histopathological diagnosis remains controversial due to the lack of reliable diagnostic and prognostic markers. Thus, according to the World Health Organization (WHO), malignant pheochromocytomas can only be defined with the presence of regional invasion or metastasis in a non-chromaffin site. The PASS was defined in 2002 by Thompson as a multiparameter scoring system to distinguish benign from malignant neoplasms [6]. This scoring system uses nine different microscopic features including: capsular invasion, vascular invasion, extension into the peri-adrenal adipose tissue, presence of large nests or diffuse growth (in >10% of tumor volume), central tumor necrosis (in the middle of large nests) or confluent necrosis, high cellularity, tumor cell spindling, cellular monotony, increased mitotic figures, atypical mitotic figures, and profound nuclear polymorphism. This scoring system is the most widely used method to define malignancy (score >4). In a study conducted by de Waily et al., the score threshold of ≥4 was confirmed to suggest the potential development of malignancy. Additionally, no neoplasia that was scored PASS <5 evolved into metastasis, and 100% of malignant pheochromocytomas had a PASS score ≥5.2.

## Conclusions

In conclusion, despite the rarity of pheochromocytomas, it is important to keep these tumors in the differential diagnosis, especially when considering a patient with refractory HTN. Any incidentaloma with appropriate clinical history, such as uncontrolled HTN, should raise the suspicion of pheochromocytoma, with or without the usual CT findings. Additionally during any operative planning, incidental discoveries should be appropriately worked up. Especially in this case it was imperative to consider this diagnosis, not only because these tumors can be malignant, but also because these tumors can significantly increase the risk of surgical mortality if untreated prior to surgery. Despite the controversial diagnostic criteria for pheochromocytomas, the PASS scoring system appears to correlate to pheochromocytoma malignancy. Those with scores higher than 4 have a high likelihood of malignancy and require adjuvant treatment.  
